# Type 1 Diabetes Patients’ Practice, Knowledge and Attitudes towards Influenza Immunization

**DOI:** 10.3390/vaccines9070707

**Published:** 2021-06-29

**Authors:** Giulia Dallagiacoma, Agnese Allora, Stefano Salvati, Giulia Cocciolo, Michele Capraro, Anna Lamberti, Sabrina Senatore, Leandro Gentile, Vincenza Gianfredi, Andrea Laurenzi, Chiara Molinari, Amelia Caretto, Marino Faccini, Carlo Signorelli, Marina Scavini, Anna Odone

**Affiliations:** 1 Department of Public Health, Experimental and Forensic Medicine, University of Pavia, 27100 Pavia, Italy; giulia.dallagiacoma01@universitadipavia.it (G.D.); leandro.gentile01@universitadipavia.it (L.G.); 2 School of Medicine, Vita-Salute San Raffaele University, 20132 Milan, Italy; allora.agnese@hsr.it (A.A.); salvati.stefano@hsr.it (S.S.); cocciolo.giulia@hsr.it (G.C.); Capraro.Michele@hsr.it (M.C.); gianfredi.vincenza@hsr.it (V.G.); signorelli.carlo@hsr.it (C.S.); 3 Agency for Health Protection of Metropolitan Area of Milan (ATS), 20121 Milan, Italy; alamberti@ats-milano.it (A.L.); Ssenatore@ats-milano.it (S.S.); MFaccini@ats-milano.it (M.F.); 4 Diabetes Research Institute, San Raffaele Hospital, 20132 Milan, Italy; laurenzi.andrea@hsr.it (A.L.); molinari.chiara@hsr.it (C.M.); caretto.amelia@hsr.it (A.C.); scavini.marina@hsr.it (M.S.)

**Keywords:** vaccination, influenza, diabetes, type 1 diabetes mellitus

## Abstract

Diabetic patients are at higher risk of developing infectious diseases and severe complications, compared to the general population. Almost no data is available in the literature on influenza immunization in people with type 1 diabetes mellitus (T1DM). As part of a broader project on immunization in diabetic patients, we conducted a cross-sectional study to: (i) report on seasonal influenza coverage rates in T1DM patients, (ii) explore knowledge, attitudes, and practices (KAPs) towards seasonal influenza in this population, and (iii) identify factors associated with vaccine uptake, including the role of family doctors and diabetologists. A survey was administered to 251 T1DM patients attending the Diabetes Clinic at San Raffaele Research Hospital in Milan, Italy and individual-level coverage data were retrieved from immunization registries. Self-reported seasonal influenza immunization coverage was 36%, which decreased to 21.7% when considering regional immunization registries, far below coverage target of 75%. More than a third (36.2%) of T1DM patients were classified as pro-vaccine, 30.7% as hesitant, 17.9% as uninformed, and 15.1% as anti-vaccine. Diabetologists resulted to be the most trusted source of information on vaccines’ benefits and risks (85.3%) and should be more actively involved in preventive interventions. Our study highlights the importance of developing tailored vaccination campaigns for people with diabetes, including hospital-based programs involving diabetes specialists.

## 1. Introduction

Diabetes is a chronic condition heavily affecting not only the lives and well-being of patients and their families but also economy and societies worldwide [1]. Being among the top 10 mortality causes in adult populations, with an estimate of four million deaths globally in 2017, diabetes is a major public health challenge [2,3]. As reported by the International Diabetes Federation (IDF), 463 million people worldwide currently live with a diagnosis of Diabetes Mellitus (DM), causing a global health expenditure of $760 billion [2,4]. This includes both direct costs, related to the treatment of diabetes and its complications, and indirect costs due to production losses of working-age individuals and premature deaths [5].

Compared to the general population, diabetic patients are at higher risk of developing infectious diseases and their severe complications, with higher hospitalization and death rates [6,7,8,9]. A recent cohort study in Canada found that patients with diabetes had a 21% increased risk of developing a new infection, compared to the general population, over a 4-year time frame [10]. Influenza is particularly important in this context, since patients with diabetes have a six-fold increased risk of hospitalization and are three times more likely to die from influenza-related complications compared to the general population [11].

National and international health authorities, including the American Diabetes Association (ADA) and the Centers for Disease Control and Prevention (CDC) Advisory Committee on Immunization Practices (ACIP) recommend seasonal influenza immunization for patients with diabetes, together with other selected immunization programs, including tetanus, pertussis, diphtheria, herpes zoster, pneumococcal, and hepatitis B vaccinations [12,13]. In Italy, according to the Italian National Vaccine Prevention Plan 2017–2019 (Piano Nazionale Prevenzione Vaccinale—PNPV), and the Standard of Care 2018 of the Italian Association of Diabetes Physicians and the Italian Diabetes Society (Standard di Cura AMD-SID 2018), both patients with diabetes, their family members and/or caregivers are offered, every year, the influenza vaccination free-of-charge, regardless of their age [14,15,16,17].

Despite the strong rationale for recommending influenza immunization to patients with diabetes [18], available data report that vaccine coverage rates remain low, and below the coverage targets set by health authorities [7,19,20]. In addition, influenza immunization coverage data are not routinely collected and monitored for this high-risk population. Recent data from Spain reported that influenza vaccine uptake among adults with diabetes was 63.8% in 2006, and it dropped to 40% in 2017 [21]. Similar situations are reported outside Europe, with influenza vaccination rates for patients with diabetes in Taiwan at 31–35% [22]. In the United States, influenza vaccination coverage among adults with diabetes was 64.8% in 2017, and even though it remained below the optimal threshold, it was higher than among patients without diabetes (43.9%) [23]. In Italy, data from a national population-based surveillance system reported that, in 2016–2019, only 28.8% of individuals with diabetes, aged 18–64, were vaccinated against seasonal influenza [24].

Factors influencing vaccine uptake in patients with diabetes are related to the availability of effective and efficient immunization programs, to the demand for immunization, and the willingness to be vaccinated [25]. In particular, factors influencing demand for influenza immunization might differ among patients with diabetes, as compared to individuals in the general population.

The current study is part of a broader project aiming to assess knowledge, attitudes, and practices (KAPs) towards influenza vaccination and its uptake among adult patients with diabetes. In particular, the current paper focuses on patients with Type 1 Diabetes Mellitus (T1DM), which are estimated to account for 5–10% of all DM cases globally [26]. General aim was to gather useful data to inform the organization of tailored immunization programs for this population. Specific objectives were: (i) to report on seasonal influenza coverage rates in T1DM patients, (ii) to explore KAPs toward seasonal influenza in this population, and (iii) to identify factors associated with vaccine uptake, with particular reference to the role played by family doctors and diabetologists.

## 2. Materials and Methods

Data sources for the current study include: (i) an ad hoc survey designed by the study group and (ii) influenza vaccine coverage data collected through the regional Immunization Information System (IIS). A survey on KAPs toward influenza immunization was designed and administered in person to a sample of adult (≥18 years old) T1DM patients attending the Diabetes Clinic at San Raffaele Research Hospital in Milan, Italy, which is nationally recognized as one of the leading centres of excellence for the study, prevention and treatment of diabetes. Interviews were conducted between 16 May 2019 and 18 February 2020. The study protocol was approved by the Ethics Committee of the San Raffaele Hospital. Consent to participate was collected from all study participants prior to data collection.

The survey tool was designed and adapted based on a previously validated questionnaire, used by the Italian National Institute of Health (Istituto Superiore di Sanità, ISS) to investigate vaccine hesitancy among parents [27].

The survey was available in digital format, on an online platform complying with the new European standards for privacy regarding data collection and storage. Surveys were administered in person by ad hoc trained members of the study group. The survey was composed of 52 items, divided into two parts. The first part collected socio-demographic data (i.e., level of education, marital status, and socio-economic status) and medical history data, including: time from disease diagnosis, current therapy, most recent glycated haemoglobin (HbA1c) value, and use of carbohydrates counting and ketones measurement. The second part of the survey was divided into three sections investigating different aspects related to vaccinations and influenza among patients with diabetes: (i) attitudes and practices towards influenza immunization, (ii) knowledge on risks and prevention of influenza, and (iii) information sources and trust in different categories of physicians. The first section focused on patients’ attitudes towards influenza vaccination and their self-reported vaccination status. Patients were asked whether they, or their family members, had been vaccinated against seasonal influenza in the past three years. In case of a negative answer, they had to specify the primary reason for non-compliance. In case of a positive answer, patients were asked the source of the recommendation and where they had been vaccinated (at home/in primary care clinics/in prevention services clinics/at the hospital). The second section aimed at investigating patients’ knowledge and awareness on: (i) higher risk of infection and infectious diseases’ complications in DM patients vs. the general population, (ii) recommended vaccinations for high-risk groups and their household contacts. The third section investigated patients’ sources of information on vaccines and immunization programs. In particular, they were asked to report the advice received by their family doctors and diabetologists, exploring any contrasting opinions received from these two healthcare professionals, as well as the level of trust towards them. Then, they were asked their perceived level of reliability of different information sources, including healthcare professionals, internet and other media, friends, and relatives. Of note, the last section of the survey explored patients’ attitudes and willingness of being vaccinated against influenza at the hospital diabetes clinic, should the service be available. Finally, 2019–2020 influenza immunization uptake data were retrieved from the regional IIS and individually linked to the survey data to derive coverage rates in the study population, limited to residents of the Lombardy region.

At the analysis stage we grouped study participants, by level of hesitancy towards influenza immunization, on the basis of a previously published classification issued by colleagues from the Italian National Institute of Health [27]. Participants were therefore identified based on their response to the question “Were you vaccinated against influenza during the last flu season?”: patients who responded they had been vaccinated were considered as pro-vaccine; patients who answered they were not vaccinated but indented to get vaccinated in the future, or who were unsure on whether or not to get vaccinated, were classified as hesitant, regardless of the reasons behind their choice. Those who did not know that a vaccine was available, recommended, or had never considered the possibility of being vaccinated were classified as uninformed, as they lacked some information in their decision-making process (specifically, they were not aware that influenza vaccine is recommended and offered to people with DM). Lastly, patients who had not been vaccinated, and declared they were not willing to get vaccinated in the future, were classified as anti-vaccine. Importantly, this classification only refers to participants’ attitude towards seasonal influenza immunization, which represents the core of this study. Knowledge, attitudes, beliefs, and sources of information on both influenza and other vaccines, along with other exposure variables of interest were compared in the four groups. Knowledge on the risk of infections, and on immunization recommendations, for patients with diabetes was categorized into three levels: poor, average, and good level of knowledge, based on a score (Supplementary Table S1) assigned to correct answers in specific questions of the survey. We conducted a descriptive analysis of survey responses using absolute frequencies, percentage distributions (categorical variables), and means with standard deviation (SD) (continuous variables), assessed determinants of hesitancy, and different roles played by family doctors and diabetologists in influencing the decision process on vaccine uptake. Statistical analysis was conducted with SPSS version 25.0 (IBM Corporation, Armonk, NY, USA).

## 3. Results

### 3.1. Socio-Demographic and Clinical Characteristics of the Study Population

Two hundred and fifty-one patients with T1DM were enrolled in the study. The characteristics of the study population are reported in Table 1. Fifty-one per cent of the study population were female, and the mean age was 35.5 years (±15.02 SD) with nearly half of the study population (42.6%) aged 18–29 years. Almost all patients (96.8%) were born in Italy. Fifty-eight per cent of study participants were single; level of education was medium (high school diploma) in 53.4% of cases, while 33.9% of the interviewed subjects had a high-level education (Bachelor’s degree or higher). Based on occupation, 14.5% reported a high and 42.2% a medium socio-economic status. Around a quarter (26.1%) of the study participants were students.

The average number of years since diagnosis of T1DM was 18.35 years (±11.36 SD), with the majority of responders (36.4%) having had the diagnosis for 6–10 years, 11.7% for less than 5 years, and 14.6% for 30 years or more. As for the clinical characteristics of our sample, the average HbA1c level was 7.24 (±0.92 SD), most of them (61%) measured their urine ketone levels in case of hyperglycaemia, and the majority of them used carbohydrate counting for estimating pre-prandial insulin bolus (71%).

### 3.2. Coverage, Practice and Attitudes towards Influenza Immunization

Table 2 reports data on attitudes and practices towards influenza immunization: 36.3% reported having been vaccinated during the last influenza season, 43.0% having been vaccinated at least once in the previous three years, and 26% reported having received the vaccination every year. Only 8.8% of patients reported that at least one family member was ever vaccinated against influenza to protect them from infection.

Overall, 36% of study participants were classified as ‘pro-vaccine’, 30% as ‘hesitant’, 17% as ‘mis-informed’, and 15% as ‘anti-vaccine’. Considering only study participants who were residents in the Lombardy region, after linking survey results with administrative data from the regional Immunization Information Systems, we report a 5.9% discrepancy between self-reported and administrative data (vaccination uptake was, respectively, 15.8% and 21.7%). Among study participants that reported having been vaccinated against influenza in the previous year, 58.9% were advised by their family doctor, 27% by their diabetologist, while only a smaller share of patients received recommendations from other healthcare professionals (4.5%), friends and family members (6.7%), or through mass media communication (2.2%). The most frequently reported vaccination sites were primary care clinics (38.5%), followed by preventive services clinics (27.5%). The most frequently reported reason for not getting vaccinated against influenza in the previous year was the idea that influenza vaccination was either “not useful” or “not necessary” (68.2%). Other reasons included lack of recommendations from physicians (12.7%), having forgotten or missed the vaccination appointment (12.7%), and reporting medical contraindications to vaccination (4.5%).

### 3.3. Knowledge and Opinions on Diabetes and Influenza Vaccination

Table 3 reports participants’ knowledge on vaccines and immunization, recommended either for the general population or for patients with diabetes. Overall, study participants were aware of the importance of vaccines in protecting collective health and did not believe in fake news. However, about one fifth of patients with diabetes were not aware of being at higher risk of infections and their complications, which increased to one fourth in the uninformed and anti-vaccine groups. Most patients (70.5%) knew that infectious diseases might have a worse clinical course in patients with diabetes, compared to individuals in the general population. Nevertheless, 61% of study participants were not aware of the recommended vaccinations available for them, and 81.7% didn’t know that the same vaccinations were recommended for their close relatives.

Knowledge score distribution showed that, overall, DMT1 patients had a good knowledge on diabetes related aspects (26% high score; 39% average score), which increased among the pro-vaccine subgroup (30% high score; 45% average score). On the contrary, uninformed and anti-vaccine subjects reported lower knowledge on this issue: a high knowledge score was reported in only 24% of uninformed and 18% of anti-vaccine patients.

Table 4 reports participants’ opinions on vaccinations. Only 15.1% of the responders reported they were afraid of adverse reactions, and 6.4% were afraid of possible long-term damage after vaccination, with similar percentages among all groups. The survey also showed that most of the responders (66.9%) felt adequately informed when deciding whether to be vaccinated or not, especially among the pro-vaccine group (83.1%). Of note, 51.1% of those classified as ‘uninformed’ felt adequately informed about vaccinations. Only 6% of the responders reported that the free-of-charge vaccinations offered by Local Health Units were too many, mostly belonging to the anti-vaccine group (50% of those who expressed this opinion).

### 3.4. Trust and Role of Information Sources

Table 5 reports patients’ trust in their family doctor and diabetologist/endocrinologist. In case of doubts about the real risks and benefits of a vaccine, 77.7% of patients would ask their family doctor and 85.3% would turn to their diabetologist, while 33.1% would ask public health practitioners in their Local Health Units. Similarly, responders trusted that a vaccine is safe if recommended by their family doctor (75.7%) or their treating diabetologists (86%). The final decision on whether to get vaccinated was influenced by the family doctor’s opinion in 59% of cases and by the treating diabetologist’s advice in 80.9% of cases.

T1DM patients who reported not having received any advice on the importance of getting vaccinated from their treating diabetologists and from their family doctors were 63.7% and 56.6%, respectively. In particular, only 14.7% of diabetologists and 23.1% of family doctors recommended their diabetic patients getting vaccinated against seasonal influenza. Of note, 11.2% found inconsistencies between different health professionals’ opinions on vaccinations. As for the existence of immunization programs recommended for their close relatives, only 11.6% of our sample had received this information from their family doctor, and 10.4% from their treating diabetologist. The immunization programmes for close relatives of diabetic patients appeared to be widely unknown, especially among the uninformed and anti-vaccine subgroups, where only 37% and 41% of participants reported to be aware of these vaccinations.

Sixty-one percent of responders reported that they usually do not use the internet to gather information on vaccinations. Among the different options available on the internet to get information on this issue, the most widely accessed by our patients were the websites of their Local Health Units (16.7%) and other institutional websites (17.5%), while 18.7% declared they usually perform a generic search on the internet.

Finally, 77.7% of study participants reported that, if possible, they would like to receive the vaccination at the Diabetes Clinic where they are followed. Most of these responders belonged to the ‘uninformed’ (41.5%) and ‘hesitant’ (29.7%) groups.

## 4. Discussion

We report on knowledge, attitudes, and practices towards influenza immunization in patients with T1DM. Almost no data are available in the literature on influenza vaccine uptake in this high-risk population. One study carried out in Spain in 2020, on a convenience sample of 300 subjects, reported a 55% influenza vaccination uptake in this population [19]. To our knowledge, no other recent studies reported influenza coverage data for patients with T1DM. Indeed, data on adherence to influenza immunization among adult patients with T1DM remains scant, both nationally and at the European level. The lack of data on influenza immunization coverage among patients with T1DM is likely the result of two main factors: first, coverage data among patients with diabetes are mainly collected and reported regardless of the type of diabetes; secondly, coverage data among high-risk groups are not routinely collected in many European countries, including Italy. In Italy, for instance, available data on immunization coverage, in patients with diabetes, come from self-reported estimates derived from nationwide surveillance systems (the PASSI project) on lifestyle, behavioural risk factors, and access to preventive programmes coordinated by the National Institute of Health (ISS) [28]. The latest available data from PASSI reports that influenza vaccination rate, in people with diabetes in 2018, not distinguishing by type of diabetes, was 28.8% [24], a point estimate lower when compared to our results.

We show a 36% influenza vaccination self-reported coverage in patients with T1DM, far below the coverage target of 75% set by the PNPV 2017–19 [15], which decreases to 21.7% when considering coverage data from regional immunization registries. Our study shows that influenza vaccination uptake among patients with T1DM is low, despite their risk of infection and serious complications being higher compared to the general population. A study, carried out in Ireland in 2012, reported coverage at 64.5% among patients with diabetes [29]. In France, between 2008 and 2011, the annual influenza vaccination uptake among people with type 2 diabetes (T2DM) ranged between 32.3% and 33.7%, in the 18–64 age group, and between 61.1% and 69.5% in people aged 65 years or older [30]. A Dutch study investigated vaccination uptake among patients with diabetes over a 5-year period, highlighting a gradual decrease from 85.1% in 2008 to 74.7% in 2013 [31].

As previously discussed, most studies investigate vaccination uptake among patients with diabetes, without accounting for the type of diabetes. This approach has some weaknesses and may lead to misleading results, as type 1 and type 2 DM have extremely different clinical presentations and affect different populations, with different preventive measures, needs, and treatment approaches. In particular, T1DM is a condition caused by autoimmune-induced pancreatic beta cells destruction, leading to absolute deficiency of insulin, while in T2DM, hyperglycaemia is a consequence of insulin-resistance [32]. T1DM is often associated to a younger onset, although nearly half of the incident cases of T1DM occur in individuals aged 30 years and older. T2DM typically affects older age groups [33], although the age of onset of T2DM has been progressively decreasing over the last two decades. Therefore, although often considered as two presentations of the same disease, T1DM and T2DM should be studied separately, taking into consideration the unique features of these conditions [34,35], that may differentially impact vaccines’ efficacy and effectiveness, as well as affect people’s attitudes towards several issues, including vaccination uptake. This is in line with the findings of a French study reporting that people with T1DM were more likely to accept seasonal influenza immunization over a 10-year period, compared to people with T2DM [36].

Our sample of T1DM patients appeared to be aware of their increased susceptibility to infectious diseases, including influenza, both in terms of increased risk of infection (59.8%) and severe complications (70.5%). These data are encouraging, especially when compared to previous studies that highlighted a lower degree of awareness among diabetic patients, regardless of the type of diabetes [37,38]. In particular, a survey conducted among patients with diabetes in Pretoria, South Africa reported that only 38.4% of participants were aware that influenza symptoms can be worse among patients with diabetes, and only 32.9% believed that influenza can cause serious complications in this group of people [37]. The same study reported that 49.7% of patients believed that the vaccine against influenza wasn’t safe, while in our population, we didn’t observe any relevant fear related to the safety of the influenza vaccine: in fact, all patients considered influenza vaccine safe, but an alarmingly large share believed it was either not useful or not necessary. Similar results were reported in a study carried out in Singapore, where 75.6% of unvaccinated diabetic patients reported that the reason behind their refusal was the perception that the vaccine was not necessary [38]. These data suggest the need for targeted educational interventions aimed at promoting the importance of prevention measures, such as influenza immunization, among this vulnerable population. People with diabetes should be aware, not only that they are at higher risk of infectious diseases and complications, as compared to the general population, but also that selected vaccinations, including influenza, are recommended and offered to them, and that evidence proved that these vaccines are effective and safe [39]. It’s widely known that recommendations from healthcare professionals are extremely important when making health-related decisions [37,40,41]. Information and advice from general practitioners and diabetes specialists play an essential role in increasing influenza vaccination uptake in people with diabetes, and studies have shown that patients with frequent hospital visits are more likely to accept seasonal influenza vaccination [36,42]. In particular, patients trust their diabetologist more than their family doctors, especially when making a decision regarding seasonal influenza vaccination (80.9% versus 50.9%, respectively). Our study also suggests that confusion arises in patients when contrasting opinions are given by different physicians or when not enough information is provided on this issue: in fact, one of the main reasons for missed vaccinations, especially among the ‘hesitant’ and ‘uninformed’ groups, was lack of information provided by their doctors. These two groups, accounting for almost half of our study population, represent an important target group for educational interventions to promote seasonal influenza immunization. ‘Hesitant’ patients require that physicians address their doubts and concerns, in order to build trust and provide reassurance [43,44]. In this context, the interaction between patients and physicians is the cornerstone of maintaining confidence in vaccination. Public Health plays a key role in this process as well [45] and should aim at communicating adequately with the population, providing information on the benefits, and on the importance, of preventive measures, especially among the most vulnerable groups of people [46]. Similarly, ‘uninformed’ people must be educated on the importance of immunization practices, through a proper information delivery system and the support from family doctors and diabetologists, who should adequately inform their patients on the importance of seasonal immunization against influenza [47,48].

Despite the lack of information received by their physicians, only 40% of our patients reported using the internet to gather information on vaccinations. The most widely consulted websites were those of Public Health institutions and healthcare providers, which are considered a reliable source of information [49]. This suggests that information provided through these channels should be tailored to meet the needs of patients, addressing possible doubts and concerns on vaccinations and providing all the information needed to build trust in immunization policies and make informed decisions [50]. Providing reliable and updated sources of information on the internet should be a priority for Public Health in the context of healthcare digitalization, as this channel is widely used to gather information on vaccinations [51,52], and it may be a useful tool to counteract vaccine hesitancy [53,54,55,56].

Our study has both strengths and limitations and provides inputs for future research. It is among the first to report on influenza vaccine uptake in T1DM patients and to investigate reasons for vaccine hesitancy in this population. It is part of a broader research project, resulting from a multi-disciplinary collaboration between experts in the fields of diabetes, public health, and health communication, conducted in a centre of excellence for diabetes care and technologies. We acknowledge the current study was conducted on a relatively small, and conveniently chosen, sample, although it is planned to be continued over the next two years to include larger numbers of patients with either T1DM or T2DM. Another limitation is the lack of local comparisons with previous KAPs and the lack of clinical data on previous hospitalizations or complications related to influenza that may have occurred in the past to these patients, which might affect their opinion and attitude towards this vaccination. A significant limitation is also the self-reported information on patients’ vaccination status and the inability to double-check it on the Italian Immunization Information System for those who were not residents in the Lombardy region. Nonetheless, surveys represent a quick and inexpensive method to collect and analyse data, and they can provide valuable results.

Despite these limitations, our study offers some important inputs for future research. In particular, what emerges is the need for routinely collected data on influenza burden in T1DM patients and for tailored information campaigns to raise awareness on the importance of vaccinations [57] not only for diabetic people but also for their close relatives. All physicians, including family doctors and diabetologists, should advocate for their patients’ health as a whole, focusing not only on disease treatment but also on disease prevention [58]. In particular, preventing influenza and influenza-related complications should be one of the top priorities among high-risk populations, including diabetic patients. Diabetologists, who appeared to be the most trusted source of information for diabetic patients, should be properly trained to be able to give all the relevant information on vaccinations to their patients. This will not only improve patients’ health and quality of life, but it will lift a burden from the National Health System and mark a Public Health success. Importantly, our study suggests the importance of planning and delivering influenza vaccinations at the hospitals and outpatient clinics where these people are routinely followed for their condition, thus facilitating their access to vaccination services. The integration of multiple strategies and interventions, targeting both patients and health professionals, are therefore required to improve seasonal influenza vaccination uptake in vulnerable populations, such as people with diabetes mellitus.

## Figures and Tables

**Table 1:** Socio-demographic and clinical characteristics of the study population, [n (%)].

Characteristics	Total	Pro-Vaccine	Hesitant	Uninformed	Anti-Vaccine
**Sex**	251 (100%)	91 (100%)	77 (100%)	45 (100%)	38 (100%)
Male	123 (49%)	38 (41.8%)	43 (55.8%)	25 (55.6%)	17 (44.7%)
Female	128 (50.9%)	53 (58.2%)	34 (44.2%)	20 (44.4%)	21 (55.3%)
**Age**	251 (100%)	91 (100%)	77 (100%)	45 (100%)	38 (100%)
18–29 years	107 (42.6%)	39 (42.9%)	37 (48%)	21 (46.7%)	10 (26.3%)
30–49 years	76 (30.3%)	25 (27.5%)	18 (23.4%)	18 (40%)	15 (39.5%)
50–64 years	52 (20.7%)	17 (18.7%)	19 (24.7%)	4 (8.9%)	12 (31.6%)
65 years and older	16 (6.4%)	10 (11%)	3 (3.9%)	2 (4.4%)	1 (2.6%)
**Country of origin**	251 (100%)	91 (100%)	77 (100%)	45 (100%)	38 (100%)
Italy	243 (96.8%)	89 (97.8%)	77 (100%)	41 (91.1%)	36 (94.7%)
not Italy	8 (3.2%)	2 (2.2%)	0 (0%)	4 (8.9%)	2 (5.3%)
**Marital status**	246 (100%)	91 (100%)	75 (100%)	43 (100%)	37 (100%)
Single	145 (58.9%)	51 (56%)	46 (61.3%)	29 (67.4%)	19 (51.4%)
Married	85 (34.6%)	33 (36.3%)	23 (30.7%)	12 (27.9%)	17 (45.9%)
Separated/Widow	16 (6.5%)	7 (7.7%)	6 (8%)	2 (4.7%))	1 (2.7%)
**Education**	251 (100%)	91 (100%)	77 (100%)	45 (100%)	38 (100%)
Low level	32 (12.7%)	15 (16.5%)	8 (10.4%)	4 (8.9%)	5 (13.2%)
Medium level	134 (53.4%)	45 (49.5%)	39 (50.6%)	28 (62.2%)	22 (57.9%)
High level	85 (33.9%)	31 (34%)	30 (39%)	13 (28.9%)	11 (28.9%)
**Occupational status**	249 (100%)	91 (100%)	76 (100%)	45 (100%)	37 (100%)
High-level income	36 (14.5%)	12 (13.2%)	15 (19.7%)	1 (2.2%)	8 (21.6%)
Medium- and low-level income	105 (42.2%)	38 (41.8%)	25 (32.9%)	23 (51.1%)	19 (51.3%)
Houswife/unemployed	25 (10%)	5 (5.5%)	10 (13.2%)	7 (15.6%)	3 (8.1%)
Student	65 (26.1%)	25 (27.5%)	20 (26.3%)	14 (31.1%)	6 (16.2%)
Retired	18 (7.2%)	11 (12.1%)	6 (7.9%)	0 (0%)	1 (2.7%)
**Years since diagnosis**	247 (100%)	88 (100%)	76 (100%)	45 (100%)	38 (100%)
less than 5	29 (11.7%)	13 (14.8%)	2 (2.6%)	10 (22.2%)	4 (10.5%)
6–10 years	90 (36.4%)	33 (37.5%)	32 (42.1%)	15 (33.3%)	10 (26.3%)
11–20 years	57 (23.1%)	17 (19.3%)	22 (28.9%)	9 (20%)	9 (23.7%)
21–30 years	35 (14.2%)	13 (14.8%)	8 (10.5%)	3 (6.7%)	11 (28.9%)
30 years or more	36 (14.6%)	12 (13.6%)	12 (15.8%)	8 (17.8%)	4 (10.5%)
**HbA1c level (mean, SD)**	7.241 (0.916)	7.155 (0.990)	7.316 (0.901)	7.231 (0.936)	7.297 (0.936)
**Ketones measurement**	251 (100%)	91 (100%)	77 (100%)	45 (100%)	38 (100%)
Yes	155 (61.7%)	61 (67%)	45 (58.4%)	28 (62.2%)	21 (55.3%)
No	74 (29.5%)	20 (22%)	27 (35.1%)	14 (31.1%)	13 (34.2%)
I don’t know what ketones are	22 (8.8%)	10 (11%)	5 (6.5%)	3 (6.7%)	4 (10.5%)
**Use of carbohydrate counting**	251 (100%)	91 (100%)	77 (100%)	45 (100%)	38 (100%)
Yes	53 (21.1%)	15 (16.5%)	17 (22.1%)	17 (37.8%)	4 (10.5%)
No	180 (71.7%)	68 (74.2%)	56 (72.7%)	26 (57.8%)	30 (78.9%)
I don’t know what it is	18 (7.2%)	8 (8.8%)	4 (5.2%)	2 (4.4%)	4 (10.5%)

**Table 2:** Practice and attitudes toward influenza immunization in our study population [n (%)].

Practice and Attitudes	Total	Pro-Vaccine	Hesitant	Uninformed	Anti-Vaccine
**Did you get vaccinated against influenza last year?**	251 (100%)	91 (100%)	77 (100%)	45 (100%)	38 (100%)
Yes	91 (36.3%)	91 (100%)			
No, and I’m not going to get vaccinated in the future	38 (15.1%)				38 (100%)
No, and I don’t know if I will get vaccinated in the future	47 (18.7%)		47 (61%)		
No, but I intend to get vaccinated in the future	30 (11.9%)		30 (39%)		
No, because I didn’t know a vaccine was available	4 (1.6%)			4 (8.9%)	
No, because I never thought about it	41 (16.3%)			41 (91.1%)	
**Who advised you to get vaccinated against influenza last year?**		89 (100%)			
Family doctor		53 (58.9%)			
Diabetologist/endocrinologist		24 (27%)			
Other health professionals		4 (4.5%)			
I read/heard it in newspapers/radio/TV		2 (2.2%)			
Family, friends, or acquaintances recommended it to me		6 (6.7%)			
**Where did you receive the influenza vaccination last year?**		91 (100%)			
At primary care clinics		35 (38.5%)			
At another doctor’s office		8 (8.8%)			
At home (family doctor/acquaintances/self-administered)		14 (15.4%)			
At preventive services clinics		25 (27.5%)			
At a preventive medicine/occupational medicine clinic		9 (9.9%)			
**What is the main reason why you didn’t receive the influenza vaccination last year?**	110 (100%)		74 (100%)		36 (100%)
Forgetfulness/inability to attend the appointment	14 (12.7%)		14 (18.9%)		
Medical contraindications to vaccination	5 (4.5%)		3 (4%)		2 (5.6%)
I was advised against it by a healthcare professional	1 (0.9%)				1 (2.8%)
Religious reasons	1 (0.9%)		1 (1.4%)		
Physicians did not recommend it to me	14 (12.7%)		13 (17.6%)		1 (2.8%)
I don’t believe that the vaccine is safe	0 (0%)				
I don’t believe that the vaccine is useful/necessary	75 (68.2%)		43 (58.1%)		32 (88.9%)
**In the last three years, how many times have you been vaccinated against influenza?**	248 (100%)	90 (100%)	76 (100%)	44 (100%)	38 (100%)
1 time	28 (11.3%)	13 (14.4%)	10 (13.2%)	3 (6.8%)	2 (5.3%)
2 times	15 (6%)	10 (11.1%)	5 (6.6%)		
3 times	65 (26.2%)	64 (71.1%)	1 (1.3%)		
I never received the vaccination in the last three years	140 (56.5%)	3 (3.3%)	60 (78.9%)	41 (93.2%)	36 (94.7%)
**Who advised you to get vaccinated against influenza in the last three years?**	105 (100%)	85 (100%)	15 (100%)	3 (100%)	2 (100%)
Family doctor	58 (55.2%)	48 (56.5%)	8 (53.3%)	1 (33.3%)	1 (50%)
Diabetologist/endocrinologist	26 (24.8%)	22 (25.9%)	3 (20%)		1 (50%)
Other health professionals	10 (9.5%)	7 (8.2%)	2 (13.3%)	1 (33.3%)	
Family, friends, or acquaintances recommended it to me	9 (8.6%)	6 (7.1%)	2 (13.3%)	1 (33.3%)	
I read/heard it in newspapers/radio/TV	2 (1.9%)	2 (2.3%)			
**Where were you vaccinated against influenza in the last three years?**	106 (100%)	86 (100%)	15 (100%)	3 (100%)	2 (100%)
At primary care clinics	43 (40.6%)	37 (43%)	4 (26.7%)	1 (33.3%)	1 (50%)
At another doctor’s office	5 (4.7%)	4 (4.6%)	1 (6.7%)		
At home (family doctor/acquaintances/self-administered)	16 (15.1%)	11 (12.8%)	3 (20%)	2 (66.7%)	
At preventive services clinics	32 (30.2%)	26 (30.2%)	6 (40%)		
At a preventive medicine/occupational medicine clinic	10 (9.4%)	8 (9.3%)	1 (6.7%)		1 (50%)
**What is the main reason why you didn’t receive the influenza vaccination in the last three years?**	136 (100%)	3 (100%)	60 (100%)	40 (100%)	34 (100%)
Forgetfulness/inability to attend the appointment	4 (2.9%)		4 (6.7%)		
Medical contraindications to vaccination	2 (1.5%)		1 (1.7%)		1 (2.9%)
I was advised against it by a healthcare professional	2 (1.5%)			1 (2.5%)	1 (2.9%)
Religious reasons	1 (0.7%)		1 (1.7%)		
Physicians did not recommend it to me	36 (26.5%)	3 (100%)	15 (25%)	17 (42.5%)	1 (2.9%)
I don’t believe that the vaccine is safe	9 (6.6%)		2 (3.3%)	3 (7.5%)	4 (11.8%)
I don’t believe that the vaccine is useful/necessary	82 (60.3%)		37 (61.7%)	18 (45%)	27 (79.4%)
**Has anyone in your family ever been vaccinated influenza because you have diabetes?**	246 (100%)	89 (100%)	74 (100%)	45 (100%)	38 (100%)
Yes	22 (8.9%)	14 (15.7%)	2 (2.7%)	3 (6.7%)	3 (7.9%)
No	224 (91.1%)	75 (84.3%)	72 (97.3%)	42 (93.3%)	35 (92.1%)

**Table 3:** Knowledge on vaccines and diabetes [n (%)].

Knowledge Items	Total	Pro-Vaccine	Hesitant	Uninformed	Anti-Vaccine
**Knowledge on vaccines in general, number of correct answers and percentage among responders**	251 (100%)	91 (100%)	77 (100%)	45 (100%)	38 (100%)
The entire community benefits from the vaccination of children and adults	238 (94.8%)	89 (97.8%)	71 (92.2%)	43 (95.6%)	35 (92.1%)
Vaccines cause autism	213 (84.9%)	79 (86.8%)	65 (84.4%)	35 (77.8%)	34 (89.5%)
Vaccination is not needed if you follow healthy lifestyles or natural remedies)	235 (93.6%)	86 (94.5%)	73 (94.8%)	43 (95.6%)	33 (86.8%)
Some vaccines are more dangerous than the infections they prevent	222 (88.4%)	82 (90.1%)	72 (93.5%)	39 (86.7%)	29 (76.3%)
Many vaccines contain mercury and other toxic substances	152 (60.6%)	61 (67%)	43 (55.8%)	30 (66.7%)	18 (47.4%)
I don’t think vaccinations are needed: the diseases they prevent are not that serious	237 (94.4%)	86 (94.5%)	74 (96.1%)	43 (95.6%)	34 (89.5%)
**Knowledge of diabetes-related aspects, number of correct answers and percentage among responders**	251 (100%)	91 (100%)	77 (100%)	45 (100%)	38 (100%)
People with diabetes are more at risk of catching some infectious diseases than the general population	150 (59.8%)	62 (68.1%)	46 (59.7%)	22 (48.9%)	20 (52.6%)
The course of some infectious diseases may be worse in patients with diabetes than in the general population	177 (70.5%)	67 (73.6%)	58 (75.3%)	25 (55.6%)	27 (71%)
In Italy there are recommended vaccinations for people with diabetes, regardless of their age	98 (39%)	42 (46.2%)	29 (37.7%)	14 (31.1%)	13 (34.2%)
Some vaccinations are recommended for those living with people with diabetes	46 (18.3%)	21 (23.1%)	14 (18.2%)	6 (13.3%)	5 (13.2%)
**Knowledge Score on diabetes related aspects (0–250)**	251 (100%)	91 (100%)	77 (100%)	45 (100%)	38 (100%)
Poor (0–75)	85 (33.9%)	22 (24.2%)	27 (35.1%)	20 (44.4%)	16 (42.1%)
Average (76–125)	100 (39.8%)	41 (45.1%)	30 (39%)	14 (31.1%)	15 (39.5%)
High (126–250)	66 (26.3%)	28 (30.8%)	20 (26%)	11 (24.4%)	7 (18.4%)

**Table 4:** Opinions about vaccinations. Data are reported as number of affirmative answers and percentage of responders [n, (%)].

Opinion Items	Total	Pro-Vaccine	Hesitant	Uninformed	Anti-Vaccine
**I am afraid of the adverse reactions that may occur immediately after vaccination**	38 (100%)	10 (26.3%)	8 (21.1%)	9 (23.7%)	11 (28.9%)
**I am afraid of the possible damage that can occur years after vaccination**	16 (100%)	4 (25%)	3 (18.8%)	4 (25%)	5 (31.3%)
**The proposal of vaccines by local health authorities is influenced by the economic interests of pharmaceutical companies**	51 (100%)	15 (29.4%)	15 (29.4%)	8 (15.7%)	13 (25.5%)
**I felt adequately informed when I made the decision on whether to get vaccinated or not**	158 (100%)	74 (46.8%)	41 (25.9%)	23 (14.6%)	20 (12.7%)
**Healthcare professionals provide information on the benefits of vaccinations but not on the related risks**	78 (100%)	28 (35.9%)	24 (30.8%)	12 (15.4%)	14 (17.9%)
**The free-of-charge vaccinations offered by preventive services clinics are too many**	15 (100%)	4 (26.7%)	2 (13.3%)	2 (13.3%)	7 (46.7%)

**Table 5:** Trust and role of information sources [n (%)].

Trust and Role of Information Sources	Family Doctor	Diabetologist/ Endocrinologist
**Trust on information sources on vaccinations [affirmative answers (n (%)]**		
Vaccinations are safe if recommended by	190 (75.7%)	216 (86%)
His/her opinion is decisive in deciding on vaccinations	148 (59%)	203 (80.9%)
In case of doubts about the risks and benefits of a vaccine, I would ask information to	195 (77.7%)	214 (85.3%)
**Role of information sources on vaccinations [affirmative answers (n (%)]**		
He/she advised me to do them all	24 (9.6%)	23 (9.2%)
He/she advised me to take the flu vaccination	58 (23.1%)	37 (14.7%)
He/she advised me not to do any vaccinations	2 (0.8%)	2 (0.8%)
He/she didn’t give me advice	142 (56.6%)	160 (63.7%)
He/she told me about recommended vaccines for my age and because I have diabetes	71 (28.3%)	64 (25.5%)
He/she informed me of the existence of recommended vaccinations for people with diabetes	80 (31.9%)	71 (28.3%)
He/she informed me of the existence of recommended vaccinations for close contacts and caregivers of people with diabetes	29 (11.6%)	26 (10.4%)
**When using the internet to gather information on vaccines, I usually consult:**		
I don’t consult websites for information on vaccines	153 (61%)	
I don’t consult any specific websites, I usually do a generic search on Google or other search engines	47 (18.7%)	
Wikipedia	11 (4.4%)	
the Website of my Region/Local Health Unit	42 (16.7%)	
Institutional websites (e.g., Ministry of Health, Higher Institute of Health, AIFA)	44 (17.5%)	
Websites/forums of/for people with diabetes	14 (5.6%)	
Websites/forums that advise against vaccinations	2 (0.8%)	
Websites/forums that promote vaccinations	4 (1.6%)	

## Data Availability

The data presented in this study are available from the corresponding author, upon reasonable request.

## Supplementary Materials

The following are available online at https://www.mdpi.com/article/10.3390/vaccines9070707/s1: Table S1: Framework of our knowledge score, based on selected questions from the administered survey.

## References

[1] World Health Organization Global Report on Diabetes. https://www.who.int/publications/i/item/9789241565257.

[2] Internation Diabetes Federation Diabetes Atlas, 9th Edition. https://www.diabetesatlas.org/en/.

[3] Mobasseri M., Shirmohammadi M., Amiri T., Vahed N., Hosseini Fard H., Ghojazadeh M. (2020). Prevalence and incidence of type 1 diabetes in the world: A systematic review and meta-analysis. Health Promot. Perspect..

[4] Bommer C., Heesemann E., Sagalova V., Manne-Goehler J., Atun R., Bärnighausen T., Vollmer S. (2017). The global economic burden of diabetes in adults aged 20–79 years: A cost-of-illness study. Lancet Diabetes Endocrinol..

[5] Bommer C., Sagalova V., Heesemann E., Manne-Goehler J., Atun R., Bärnighausen T., Davies J., Vollmer S. (2018). Global Economic Burden of Diabetes in Adults: Projections From 2015 to 2030. Diabetes Care.

[6] SID, AMD, SItI, FIMMG, SIMG (2018). Vaccinazioni raccomandate nel paziente diabetico adulto. JAMD.

[7] Alves C., Casqueiro J., Casqueiro J. (2012). Infections in patients with diabetes mellitus: A review of pathogenesis. Indian J. Endocrinol. Metab..

[8] Carey I.M., Critchley J.A., DeWilde S., Harris T., Hosking F.J., Cook D.G. (2018). Risk of Infection in Type 1 and Type 2 Diabetes Compared With the General Population: A Matched Cohort Study. Diabetes Care.

[9] Abu-Ashour W., Twells L.K., Valcour J.E., Gamble J.-M. (2018). Diabetes and the occurrence of infection in primary care: A matched cohort study. BMC Infect. Dis..

[10] Lau D., Eurich D.T., Majumdar S.R., Katz A., Johnson J.A. (2014). Working-age adults with diabetes experience greater susceptibility to seasonal influenza: A population-based cohort study. Diabetologia.

[11] Basevi V., Di Mario S., Morciano C., Nonino F., Magrini N. (2011). Comment on: American Diabetes Association. Standards of Medical Care in Diabetes—2011. Diabetes Care.

[12] American Diabetes Association (2011). Standards of medical care in diabetes—2011. Diabetes Care.

[13] CDC Pinkbook Course Book: Epidemiology of Vaccine Preventable Diseases. https://www.cdc.gov/vaccines/pubs/pinkbook/index.html.

[14] American Diabetes Association (2020). Introduction: Standards of Medical Care in Diabetes—2020. Diabetes Care.

[15] Ministero della Salute Piano Nazionale Prevenzione Vaccinale PNPV 2017–2019. http://www.salute.gov.it/portale/vaccinazioni/dettaglioContenutiVaccinazioni.jsp?lingua=italiano&id=4828&area=vaccinazioni&menu=vuoto.

[16] Di Bartolo P., Mannino D., Alti E., Sesti G., Purrello F., Sessa A., Icardi G., Fausto F. (2018). Consensus Statement Intersocietaria: Vaccinazioni Raccomandate Nel Paziente Diabetico Adulto. Il Diabete.

[17] Centers for Disease Control and Prevention Flu and People with Diabetes. https://www.cdc.gov/flu/highrisk/diabetes.htm.

[18] Goeijenbier M., van Sloten T.T., Slobbe L., Mathieu C., van Genderen P., Beyer W.E.P., Osterhaus A.D.M.E. (2017). Benefits of flu vaccination for persons with diabetes mellitus: A review. Vaccine.

[19] Moreno-Fernández J., García-Seco J.A., Rodrigo E.M.O., Segura A.M.S., García-Seco F., Muñoz-Rodríguez J.R. (2019). Vaccination adherence to influenza, pneumococcal and hepatitis B virus in adult type 1 diabetes mellitus patients. Prim. Care Diabetes.

[20] Shin H.-Y., Chung J.H., Hwang H.-J., Kim T.H. (2018). Factors influencing on influenza vaccination and its trends of coverage in patients with diabetes in Korea: A population-based cross-sectional study. Vaccine.

[21] Alvarez C.E., Clichici L., Patricia Guzmán-Libreros A., Navarro-Francés M., Ena J. (2017). Survey of vaccination practices in patients with diabetes: A report examining patient and provider perceptions and barriers. J. Clin. Transl. Endocrinol..

[22] Yu M.-C., Chou Y.-L., Lee P.-L., Yang Y.-C., Chen K.-T. (2014). Influenza vaccination coverage and factors affecting adherence to influenza vaccination among patients with diabetes in Taiwan. Hum. Vaccines Immunother..

[23] Hung M.-C., Lu P.-J., Srivastav A., Cheng Y.J., Williams W.W. (2020). Influenza vaccination coverage among adults with diabetes, United States, 2007–2008 through 2017–18 seasons. Vaccine.

[24] EpiCentro & Istituto Superiore di Sanità Vaccinazione Antinfluenzale: Dati Sorveglianza Passi 2015–2018. https://www.epicentro.iss.it/passi/dati/VaccinazioneAntinfluenzale.

[25] Ye L., Fang T., Cui J., Zhu G., Ma R., Sun Y., Li P., Li H., Dong H., Xu G. (2021). The intentions to get vaccinated against influenza and actual vaccine uptake among diabetic patients in Ningbo, China: Identifying motivators and barriers. Hum. Vaccines Immunother..

[26] Maahs D.M., West N.A., Lawrence J.M., Mayer-Davis E.J. (2010). Epidemiology of type 1 diabetes. Endocrinol. Metab. Clin. N. Am..

[27] Giambi C., Fabiani M., D’Ancona F., Ferrara L., Fiacchini D., Gallo T., Martinelli D., Pascucci M.G., Prato R., Filia A. (2018). Parental vaccine hesitancy in Italy—Results from a national survey. Vaccine.

[28] EpiCentro & Istituto Superiore di Sanità Progressi Delle Aziende Sanitarie per la Salute in Italia: La Sorveglianza Passi. https://www.epicentro.iss.it/passi/.

[29] Clancy U., Moran I., Tuthill A. (2012). Prevalence and predictors of influenza and pneumococcal vaccine uptake in patients with diabetes. Ir. Med. J..

[30] Verger P., Cortaredona S., Pulcini C., Casanova L., Peretti-Watel P., Launay O. (2015). Characteristics of patients and physicians correlated with regular influenza vaccination in patients treated for type 2 diabetes: A follow-up study from 2008 to 2011 in southeastern France. Clin. Microbiol. Infect..

[31] Tacken M.A.J.B., Jansen B., Mulder J., Campbell S.M., Braspenning J.C.C. (2015). Dutch influenza vaccination rate drops for fifth consecutive year. Vaccine.

[32] Schmidt A.M. (2018). Highlighting Diabetes Mellitus. Arterioscler. Thromb. Vasc. Biol..

[33] Guthrie R.A., Guthrie D.W. (2004). Pathophysiology of Diabetes Mellitus. Crit. Care Nurs. Q..

[34] Tuomi T. (2005). Type 1 and type 2 diabetes: What do they have in common?. Diabetes.

[35] American Diabetes Association (2011). Diagnosis and classification of diabetes mellitus. Diabetes Care.

[36] Bocquier A., Cortaredona S., Fressard L., Galtier F., Verger P. (2020). Seasonal influenza vaccination among people with diabetes: Influence of patients’ characteristics and healthcare use on behavioral changes. Hum. Vaccines Immunother..

[37] Olatunbosun O.D., Esterhuizen T.M., Wiysonge C.S. (2017). A cross sectional survey to evaluate knowledge, attitudes and practices regarding seasonal influenza and influenza vaccination among diabetics in Pretoria, South Africa. Vaccine.

[38] Tan E.K., Lim L.H., Teoh Y.L., Ong G., Bock H.L. (2010). Influenza and seasonal influenza vaccination among diabetics in Singapore: Knowledge, attitudes and practices. Singap. Med. J..

[39] Dos Santos G., Tahrat H., Bekkat-Berkani R. (2018). Immunogenicity, safety, and effectiveness of seasonal influenza vaccination in patients with diabetes mellitus: A systematic review. Hum. Vaccines Immunother..

[40] Szucs T.D., Wahle K., Müller D. (2006). Influenza vaccination in Germany. A population-based cross-sectional analysis of three seasons between 2002 and 2005. Med. Klin..

[41] Mangtani P., Breeze E., Stirling S., Hanciles S., Kovats S., Fletcher A. (2006). Cross-sectional survey of older peoples’ views related to influenza vaccine uptake. BMC Public Health.

[42] Censis Indagine Censis 2017: La Vaccinazione Antinfluenzale nel Nuovo Assetto Demografico. https://www.youtube.com/watch?v=r59RtCVnWEk.

[43] Jarrett C., Wilson R., O’Leary M., Eckersberger E., Larson H.J., Eskola J., Liang X., Chaudhuri M., Dube E., Gellin B. (2015). Strategies for addressing vaccine hesitancy—A systematic review. Vaccine.

[44] Shen S., Dubey V. (2019). Addressing vaccine hesitancy. Can. Fam. Physician.

[45] Signorelli C., Odone A., Ricciardi W., Lorenzin B. (2019). The social responsibility of public health: Italy’s lesson on vaccine hesitancy. Eur. J. Public Health.

[46] Gianfredi V., Grisci C., Nucci D., Parisi V., Moretti M. (2018). Communication in health. Recenti Prog. Med..

[47] Pluviano S., Watt C., Ragazzini G., Della Sala S. (2019). Parents’ beliefs in misinformation about vaccines are strengthened by pro-vaccine campaigns. Cogn. Process..

[48] Durant W. (2019). How do we respond to the challenge of vaccine misinformation?. Perspect. Public Health.

[49] Gianfredi V. (2019). Trust and reputation management, branding, social media management nelle organizzazioni sanitarie: Sfide e opportunità per la comunità igienistica italiana. J. Prev. Med. Hyg..

[50] Odone A., Ferrari A., Spagnoli F., Visciarelli S., Shefer A., Pasquarella C., Signorelli C. (2015). Effectiveness of interventions that apply new media to improve vaccine uptake and vaccine coverage: A systematic review. Hum. Vaccines Immunother..

[51] Stahl J.P., Cohen R., Denis F., Gaudelus J., Martinot A., Lery T., Lepetit H. (2016). The impact of the web and social networks on vaccination. New challenges and opportunities offered to fight against vaccine hesitancy. Med. Mal. Infect..

[52] Bragazzi N.L., Barberis I., Rosselli R., Gianfredi V., Nucci D., Moretti M., Salvatori T., Martucci G., Martini M. (2017). How often people google for vaccination: Qualitative and quantitative insights from a systematic search of the web-based activities using Google Trends. Hum. Vaccines Immunother..

[53] Ferro A., Odone A., Siddu A., Colucci M., Anello P., Longone M., Marcon E., Castiglia P., Bonanni P., Signorelli C. (2015). Monitoring the web to support vaccine coverage: Results of two years of the portal VaccinarSì. Epidemiol. Prev..

[54] Odone A., Tramutola V., Morgado M., Signorelli C. (2018). Immunization and media coverage in Italy: An eleven-year analysis (2007–17). Hum. Vaccines Immunother..

[55] Gianfredi V., Moretti M., Lopalco P.L. (2019). Countering vaccine hesitancy through immunization information systems, a narrative review. Hum. Vaccines Immunother..

[56] Odone A., Signorelli C. (2017). When vaccine hesitancy makes headlines. Vaccine.

[57] Burioni R., Odone A., Signorelli C. (2018). Lessons from Italy’s policy shift on immunization. Nature.

[58] Cheloni R., Gandolfi S.A., Signorelli C., Odone A. (2019). Global prevalence of diabetic retinopathy: Protocol for a systematic review and meta-analysis. BMJ Open.

