# Supplementary material for: Type 1 Diabetes Patients’ Practice, Knowledge and Attitudes towards Influenza Immunization
Source: Vaccines (Basel). 2021 Jun 29;9(7):707. doi: 10.3390/vaccines9070707 (PMC8310009; doi:10.3390/vaccines9070707)
Supplement: Supplementary file 1 [file vaccines-09-00707-s001.zip › vaccines-1249341-supplementary.pdf]

Table S1. Framework of our knowledge score, based on selected questions from the administered survey.

	Score Assigned to Correct Answer
**General questions**	
People with diabetes are at higher risk of developing infectious diseases compared to the general population.	50
The course of some infectious diseases may be worse in patients with diabetes compared to the general population.	50
In Italy there are recommended vaccinations for people with diabetes, regardless of their age.	25
Some vaccinations are recommended for close contacts and caregivers of people with diabetes.	25
**What vaccinations do you think are recommended by the Ministry of Health and offered free of charge for people with diabetes, regardless of their age?**	
Chicken pox vaccine	10
Herpes Zoster vaccine	10
Pneumococcal vaccine	10
Meningococcal type B vaccine	10
Meningococcal type C / ACWY vaccine	10
Influenza vaccine	10
Measles / mumps / rubella vaccine	10
Hepatitis A vaccine	10
Tuberculosis vaccine	10
Human Papillomavirus vaccine	10
**Total**	**0–250**
